# Predictors of the experience of a Cytosponge test: analysis of patient survey data from the BEST3 trial

**DOI:** 10.1186/s12876-022-02630-1

**Published:** 2023-01-10

**Authors:** Bhagabati Ghimire, Rebecca Landy, Roberta Maroni, Samuel G. Smith, Irene Debiram-Beecham, Peter D. Sasieni, Rebecca C. Fitzgerald, Greg Rubin, Fiona M. Walter, Jo Waller, BEST3 Consortium, Stephen Attwood, Max Parmar, Brendan Delaney, John de Caestecker, Wendy Atkin, Allan Hackshaw, Charles van Heyningen, Tim Underwood, Alberto Stella, Charlotte Saxby, Attila Lorincz, Naomi Turnbull, Jamie Doorbar, Georgia Mannion-Krase, Irene Kaimi, Mary Kasanicki, Stephen Kelleher, Louise Stockley, Tracy Assari, Sonakshi Kadyan, Victoria Hollamby, Katie Edwards, Helen MacDonald, Viv Shaw, Heather Leishman, Holly Roper, Kate McCloskey, Helen Jung, Alex Phillips, Gosia Masjak-Newman, Kim Fell, Helen Collins, Olga Zolle, Pauline McGlone, Tania Crabb, Lauren Merrin, Martine Cross, Alex Jones, Tom Simpson, Emma Murray, Andrew Perugia, Marie Thompson, Jen Dumbleton, Monique Morar, Nadia Frowd, Antonia Hardcastle, Debbie Carmichael, Fiona Maxton, Frances Farnworth, Elaine Baddeley, Judith Offman

**Affiliations:** 1 Cancer Prevention Group, School of Cancer and Pharmaceutical Sciences, King’s College London, London, UK; 2 Present Address: Department of Health Sciences, College of Health, Medicine and Life Sciences, Brunel University London, London, UK; 3 Division of Cancer Epidemiology and Genetics, Department of Health and Human Services, National Cancer Institute, National Institutes of Health, Bethesda, MD USA; 4 Cancer Research UK and King’s College London Cancer Prevention Trials Unit, Cancer Prevention Group, School of Cancer and Pharmaceutical Sciences, King’s College London, London, UK; 5 Leeds Institute of Health Sciences, University of Leeds, Leeds, UK; 6 Early Cancer Institute, Department of Oncology, University of Cambridge, Cambridge, UK; 7 Cambridge University Hospitals NHS Foundation Trust, Cambridge, UK; 8 Population Health Sciences Institute, Newcastle University, 5th Floor, Ridley 1, Newcastle Upon Tyne, UK; 9 The Primary Care Unit, Department of Public Health and Primary Care, University of Cambridge, Cambridge, UK; 10 Centre for Prevention, Detection and Diagnosis, Wolfson Institute of Population Health, Queen Mary University of London, Charterhouse Square, London, EC1M 6BQ UK; 11 BEST3 Trial Team NIHR, Clinical Research Networks, London, UK

**Keywords:** Barrett’s oesophagus, Cytosponge, Patient experience, Inventory to assess patient satisfaction

## Abstract

**Background:**

The Cytosponge is a cell-collection device, which, coupled with a test for trefoil factor 3 (TFF3), can be used to diagnose Barrett’s oesophagus, a precursor condition to oesophageal adenocarcinoma. BEST3, a large pragmatic, randomised, controlled trial, investigated whether offering the Cytosponge-TFF3 test would increase detection of Barrett’s. Overall, participants reported mostly positive experiences. This study reports the factors associated with the least positive experience.

**Methods:**

Patient experience was assessed using the Inventory to Assess Patient Satisfaction (IAPS), a 22-item questionnaire, completed 7–14 days after the Cytosponge test.

**Study cohort:**

All BEST3 participants who answered ≥ 15 items of the IAPS (N = 1458).

**Statistical analysis:**

A mean IAPS score between 1 and 5 (5 indicates most negative experience) was calculated for each individual. ‘Least positive’ experience was defined according to the 90th percentile. 167 (11.4%) individuals with a mean IAPS score of ≥ 2.32 were included in the ‘least positive’ category and compared with the rest of the cohort. Eleven patient characteristics and one procedure-specific factor were assessed as potential predictors of the least positive experience. Multivariable logistic regression analysis using backwards selection was conducted to identify factors independently associated with the least positive experience and with failed swallow at first attempt, one of the strongest predictors of least positive experience.

**Results:**

The majority of responders had a positive experience, with an overall median IAPS score of 1.7 (IQR 1.5–2.1). High (OR = 3.01, 95% CI 2.03–4.46, *p* < 0.001) or very high (OR = 4.56, 95% CI 2.71–7.66, *p* < 0.001) anxiety (relative to low/normal anxiety) and a failed swallow at the first attempt (OR = 3.37, 95% CI 2.14–5.30, *p* < 0.001) were highly significant predictors of the least positive patient experience in multivariable analyses. Additionally, sex (*p* = 0.036), height (*p* = 0.032), alcohol intake (*p* = 0.011) and education level (*p* = 0.036) were identified as statistically significant predictors.

**Conclusion:**

We have identified factors which predict patient experience. Identifying anxiety ahead of the procedure and discussing particular concerns with patients or giving them tips to help with swallowing the capsule might help improve their experience.

*Trial registration* ISRCTN68382401.

**Supplementary Information:**

The online version contains supplementary material available at 10.1186/s12876-022-02630-1.

## Background

Around 9200 oesophageal cancers are diagnosed in the UK every year [1]. 10 year survival of this cancer is very low at just 12%, mostly due to late diagnosis [1]. In 2018, in the UK, roughly two-thirds of newly diagnosed oesophageal cancers were oesophageal adenocarcinomas (OAC), one of the two main histological subtypes [2]. Gastro-oesophageal reflux disease (GORD), smoking and obesity, in addition to sex and age, are the main risk factors for OAC [3]. Barrett’s oesophagus, which occurs in 3–6% of individuals with GORD, is a precursor condition to OAC [4]. Patients with Barrett’s are recommended to undergo regular endoscopic surveillance for pre-cancerous or cancerous lesions so that cancer can be prevented or diagnosed early, when it is easier to treat [5]. However, as screening all GORD patients for Barrett’s using endoscopy is not feasible, only a small proportion of patients with Barrett’s are diagnosed and most OACs present de novo [5, 6].

The Cytosponge is a non-endoscopic cell-collection device, which, coupled with a laboratory test for the biomarker trefoil factor 3 (TFF3), can be used to diagnose intestinal metaplasia, which is a hallmark of Barrett’s oesophagus [7]. Prior to BEST3, this device had been shown to be safe and acceptable to more than 2000 patients in two studies [7, 8]. We carried out BEST3, a large pragmatic, randomised, controlled trial, to investigate whether offering the Cytosponge-TFF3 test to GORD patients would increase detection of Barrett’s [9]. Invitation to the Cytosponge-TFF3 test (N = 6834) led to increased Barrett’s diagnosis compared to usual care (adjusted RR 10.6, 95% CI 6.0–18.8) [10]. The BEST3 trial protocol and Cytosponge procedure are described in more details in Offman et al*.* [9]. In brief, the Cytosponge is a 3 cm diameter mesh sphere on a string compressed within a gelatine capsule. The patient swallows the capsule while holding onto the string. After 5 min, the capsule dissolves and the Cytosponge expands. The nurse quickly pulls the Cytosponge from the stomach to the oesophagus and mouth using the string, thus collecting cells from the whole of the oesophagus and oropharynx. These samples are then assessed for Barrett’s. Overall, the patient experience in this study was very good and only 142 (9%) participants who successfully swallowed the Cytosponge (N = 1654) reported an adverse event: one serious adverse event, which patients were warned about at consent, where the sponge detached from the thread and required endoscopic retrieval, and a range of predominately gastro-intestinal adverse events, with a sore throat occurring most commonly in 63 (4%) participants followed by indigestion/reflux (N = 19%) and oesophageal or gastric pain (N = 15%).

Good patient experience is a key aspect of providing high-quality medical care and is associated with higher levels of adherence to recommended prevention and treatment plans, and better clinical effectiveness and outcomes [11]. Specifically, patient experience has been linked with re-attendance in both breast and cervical cancer screening, where, for example, pain experienced during the procedure or fear of pain was linked with lower re-attendance [12–14]. In addition to the experience of the procedure itself, practical barriers, such as availability of and distance to the appointment, can also result in non-attendance.

Although patient experience was good overall in the BEST3 trial, some participants reported negative experiences of some aspects of the procedure, particularly in relation to the swallowing and retrieval of the device [15]. This study reports the patient- and procedure-related factors associated with the least positive experience. The overall aim was to identify modifiable factors and patient groups most likely to have the least positive experience so that any potential issues can be addressed during the Cytosponge appointment. These findings will then help to inform the development of guidelines or interventions to minimise negative experience of the Cytosponge procedure.

## Methods

We report an exploratory analysis of patient experience data collected as part of the BEST3 trial, a multicentre, pragmatic, randomised controlled trial that took place in 109 socio-demographically diverse general practice clinics in England in 2017–2019. The design of the BEST3 trial is described in more detail elsewhere [9, 15]. Patients in the intervention arm attending a Cytosponge appointment were asked to complete a baseline questionnaire before undergoing the procedure (see *Measures* for details). They were then asked to swallow the Cytosponge with water. If they failed to swallow the capsule at the first attempt, they were given a further attempt; if they failed to or refused to swallow at the second attempt, they were considered as patients unsuccessfully swallowing the Cytosponge. Those achieving a successful Cytosponge swallow received a follow-up questionnaire to fill in 7–14 days after the procedure.

### Study cohort

**Fig. 1:**
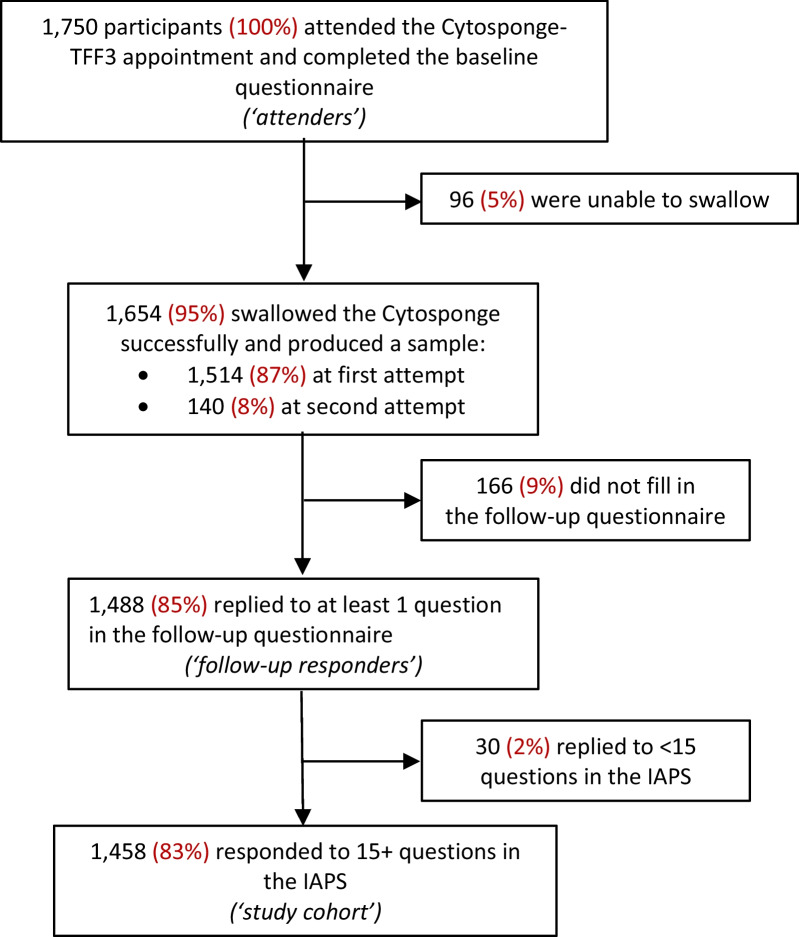
Trial flowchart for the IAPS analysis of the BEST3 trial

Overall, 1750 patients attended a Cytosponge appointment and completed the baseline questionnaire (Fig. 1). Of these, 1488 participants (85%) both successfully swallowed the Cytosponge and completed the follow-up questionnaire. Participants completing and not completing the follow-up questionnaire were compared previously: there were small but statistically significant differences in the distribution of age groups, waist-hip ratio categories and comorbidity status [15]. The study cohort for this analysis are the 1458 follow-up responders who answered 15 items or more (at least 68% of the 22 questions) of the Inventory to Assess Patient Satisfaction (IAPS).

### Measures

The outcome of interest in this analysis was the mean score on the IAPS, completed by BEST3 participants as part of their follow-up questionnaire. The IAPS consists of 22 items, both positively and negatively worded, addressing all aspects of the Cytosponge-TFF3 procedure and was adapted from a study on flexible sigmoidoscopy screening [15, 16]. Items were coded from 1 = ‘Strongly agree’ to 5 = ‘Strongly disagree’ and were reverse scored for negatively worded questions. Item scores were summed and divided by the number of questions answered to obtain a mean score between 1 and 5 for each individual, with 1 representing the most positive experience and higher scores indicating lower patient satisfaction. As the internal reliability of the scale was good (Cronbach’s alpha = 0.83), a mean score was calculated for all participants who completed 15 or more of the 22 (> 68%) items. Responses to individual items have been reported elsewhere [15].

#### Dependent variable

Patient experience was dichotomised to form a binary outcome. In the 1458 participants with 15 or more responses to the IAPS questionnaire, the ‘least positive’ experience was defined as having a mean IAPS score of ≥ 2.32, corresponding to the 90th percentile. As the 90th percentile fell within a group of individuals, 1291 patients were included in the 'positive' (89%) and 167 in the 'least positive' (11%) experience categories.

#### Independent variables

Eleven patient characteristics and one procedure specific factor were assessed as potential predictors of the least positive experience and included in the analysis (see Table 1), with some of the continuous covariates analysed as categorical variables. Demographic and anthropometric characteristics analysed were: sex (female, male); age (50–59, 60–69, 70–79, 80+ years); height (≤ 160 cm, > 160–170 cm, > 170–180 cm, > 180 cm); body mass index (BMI; < 25.0 kg/m^2^, 25.0–29.9 kg/m^2^, 30.0 and over kg/m^2^); educational level (school up to 15–16 years, college or vocational school, professional training beyond college, university graduate or postgraduate degree); deprivation quintiles based on the Index of Multiple Deprivation (IMD) of the participants’ general practice; smoking status (ever, never); alcohol intake (none, occasional, weekends only, daily or most days) and presence of comorbidities (no, yes).

**Table 1:** Patient characteristics for the two Cytosponge experience subgroups (n = 1458), and univariable and multivariable logistic regression analysis examining predictors of least positive experience (n = 1443 in adjusted model)

Variables	Patient experience group, n (%)		Univariable model		Multivariable model	
	Most positive (lowest 89 percent, n = 1291)	Least positive (highest 11 percent, n = 167)	Unadjusted odds ratio (95% CI)	*p*-value*	Adjusted odds ratio (95% CI)	*p*-value*
*Sex*						
Female	662 (51.3)	98 (58.7)	1	0.072	1	0.036
Male	629 (48.7)	69 (41.3)	0.74 (0.53–1.03)		0.56 (0.33–0.96)	
*Age (years)*						
50–59	241 (18.7)	41 (24.6)	1	0.186		
60–69	429 (33.2)	59 (35.3)	0.81 (0.53–1.24)			
70–79	505 (39.1)	55 (32.9)	0.64 (0.42–0.99)			
80 and above	116 (9.0)	12 (7.2)	0.61 (0.31–1.20)			
*Height*						
Up to 160 cm	326 (25.3)	48 (28.7)	1	0.473	1	0.032
> 160–170 cm	486 (37.7)	53 (31.7)	0.74 (0.49–1.12)		0.85 (0.54–1.33)	
> 170–180 cm	359 (27.8)	48 (28.7)	0.91 (0.59–1.39)		1.76 (0.94–3.28)	
> 180 cm	119 (9.2)	18 (10.8)	1.03 (0.57–1.84)		2.18 (0.97–4.86)	
Missing	1 (0.1)					
*BMI*						
Underweight/normal weight (< 25.0)	274 (21.2)	30 (18.0)	1	0.279		
Overweight (25.0–29.9)	569 (44.1)	69 (41.3)	1.11 (0.70–1.74)			
Obese (30 and over)	447 (34.7)	68 (40.7)	1.39 (0.88–2.19)			
Missing	1 (0.1)					
*Deprivation level (IMD quintiles)*						
1—most deprived	140 (10.8)	16 (9.6)	1	0.256		
2	146 (11.3)	15 (9.0)	0.90 (0.43–1.89)			
3	307 (23.8)	31 (18.6)	0.88 (0.47–1.67)			
4	346 (26.8)	48 (28.7)	1.21 (0.67–2.21)			
5—least deprived	352 (27.3)	57 (34.1)	1.42 (0.79–2.56)			
*Ever smoked*						
No	526 (40.7)	81 (48.5)	1	0.061		
Yes	761 (59.0)	86 (51.5)	0.73 (0.53–1.01)			
Missing	4 (0.3)					
*Alcohol intake*						
None	277 (21.5)	29 (17.4)	1	0.080	1	0.011
Occasional (once or twice per month)	342 (26.5)	34 (20.4)	0.95 (0.56–1.60)		1.04 (0.60–1.80)	
Weekends only	354 (27.4)	51 (30.5)	1.38 (0.85–2.23)		1.47 (0.88–2.46)	
Daily/most days	317 (24.6)	53 (31.7)	1.60 (0.99–2.58)		1.82 (1.08–3.08)	
Missing	1 (0.1)					
*Education level*						
School up to 15–16 years of age	524 (40.6)	62 (37.1)	0.72 (0.49–1.05)	0.119	0.64 (0.42–0.97)	0.036
College or vocational school	404 (31.3)	45 (27.0)	0.68 (0.45–1.02)		0.60 (0.38–0.92)	
Professional training, university, or postgraduate degree	352 (27.3)	58 (34.7)	1		1	
Missing	11 (0.9)	2 (1.2)				
*Comorbidities*						
No	155 (12.0)	24 (14.4)	1	0.382		
Yes	1136 (88.0)	143 (85.6)	0.81 (0.51–1.29)			
*GORD impact scale (week before appointment)*						
1 (low impact)	507 (39.3)	64 (38.3)	1	0.813		
> 1 (high impact)	784 (60.7)	103 (61.7)	1.04 (0.75–1.45)			
*State trait anxiety inventory (STAI-6) score*						
Low/normal (20–36)	901 (69.8)	73 (43.7)	1	< 0.001	1	< 0.001
High (37–49)	294 (22.8)	61 (36.5)	2.56 (1.78–3.69)		2.82 (1.92–4.13)	
Very high (50–80)	96 (7.4)	33 (19.8)	4.24 (2.67–6.73)		4.50 (2.71–7.48)	
*Failed swallow at first attempt*						
No	1188 (92.0)	131 (78.4)	1	< 0.001	1	< 0.001
Yes	103 (8.0)	36 (21.6)	3.17 (2.08–4.82)		3.45 (2.21–5.38)	

Sex, height, alcohol intake, education level, STAI-6 score and unsuccessful swallow at first attempt were included in the multivariable model based on stepwise backwards selection

*BMI* Body mass index; *IMD* Indices of deprivation; *GORD* Gastroesophageal reflux disease

*p-values: test for trend used for age, height, BMI, IMD quintile, alcohol intake, education and STAI-6

The Gastro-oesophageal Reflux Disease Impact Scale [17], consisting of nine items, was used to assess the impact of reflux symptoms in the week before the appointment. A total score was calculated by summing the individual item scores on a four-point ordinal scale (1 = ‘never’, 2 = ‘sometimes’, 3 = ‘often’, 4 = ‘daily’), and scaling the result by the number of questions answered. Final scores ranged from 1 to 4 (with 4 indicating greatest impact) and were categorised into two groups for analysis: low impact (final score = 1) and high impact (final score > 1).

Baseline anxiety immediately prior to the Cytosponge procedure was assessed using the short-form State-Trait Anxiety Inventory (STAI-6) [18]. Summed scores on the six items (each scored 1–4 and reversely scored where appropriate) were scaled to the standard STAI range of 20–80. The score was divided into three categories for analysis: low/normal (20–36), high (37–49) and very high (50–80), based on cut-offs used in other studies [7, 19].

The only procedure-specific factor included in the analysis was whether the first swallow attempt was failed (no, yes).

### Statistical analysis

Patient experience was dichotomised to form a binary outcome, as previously described. The median IAPS score and interquartile range (IQR) were calculated for all participants of the study cohort and both patient experience groups. The distribution of participants’ IAPS scores are presented in a histogram. The distributions of patient characteristics between the two groups were calculated and compared by univariable logistic regression. A multivariable logistic regression analysis to identify variables predictive of the least positive experience was conducted using backwards stepwise selection. Furthermore, univariable and multivariable regression analysis was carried out to identify any predictors of having a failed swallow at first attempt. We also used backwards stepwise selection for this multivariable regression. *p*-values for age, height, BMI, IMD quintile, alcohol intake, education and STAI-6 were calculated using a test for trend. Statistical significance was based on a two-sided test with an α-value of 5%.

## Results

Of the 1488 follow-up responders, 1458 answered 15 or more questions and 1288 answered all 22 questions of the IAPS (see Trial flowchart in Fig. 1). The vast majority of these responders had a positive experience: on a scale of 1–5, where a higher score indicates poorer experience, the overall median IAPS score was 1.7 (IQR 1.5–2.1). The median IAPS score in the positive experience group was 1.7 (IQR 1.4–1.9), and for the least positive experience group was 2.5 (IQR 2.4–2.6). The distribution of mean IAPS score for each participant is shown in Additional file 1: Fig. S1. Only 4.7% had a score above 2.5 and 0.5% over 3.

Table 1 shows the patient characteristics for the two subgroups of participants and results from the univariable and multivariable logistic regression analysis. Two variables were statistically significant predictors of having the least positive experience in univariable analyses: STAI-6 score and failed swallow at first attempt. A greater proportion of the least positive experience group reported very high anxiety (20%, 33/167) compared with the rest of the sample (7%, 96/1291). Individuals with high and very high levels of anxiety had odds ratios of 2.56 (95% CI 1.78–3.69) and 4.24 (95% CI 2.67–6.73), respectively, for having the least positive experience relative to those with low/normal anxiety. Over twice as many participants in the negative experience group had a failed swallow on the first attempt (22%, 36/167, compared with 8%, 103/1291, in the rest of the cohort), with these individuals having over three times the odds of having the least positive experience (OR 3.17, 95% CI 2.08–4.82) relative to individuals who swallowed the Cytosponge on the first attempt.

Six variables were found to be significant predictors of the least positive patient experience in multivariable analyses following backwards selection (Table 1). The most significant associations were found for anxiety and unsuccessful swallow at first attempt. Participants with high or very high levels of anxiety had greater odds of the least positive experience compared to participants with low/normal anxiety levels (OR 2.82, 95% CI 1.92–4.13 and OR 4.50, 95% CI 2.71–7.48 for the ‘high’ and ‘very high’ groups, respectively, *p* for trend < 0.001). Similarly, not being able to swallow the Cytosponge at the first attempt was also significantly associated with greater odds of the least positive experience (OR 3.45, 95% CI 2.21–5.38, *p* < 0.001). Sex, height, alcohol intake and education level were also significantly associated having *p*-values between 0.01 and 0.04 and confidence intervals that came closer to 1. Individuals who drank alcohol daily or on most days had increased odds of being in the least positive experience group (OR 1.82, 95% CI 1.08–3.08, *p* for trend = 0.011) relative to individuals who never drank alcohol. Individuals with school up to 15–16 years of age or with a college or vocational school level of education had lower odds of having the least positive experience relative to individuals who were university graduates (OR 0.64, 95% CI 0.42–0.97 and 0.60, 95% CI 0.38–0.92, respectively *p* for trend = 0.036). There was also a statistically significant trend of increasing odds with increased height (> 180 cm: OR 2.18, 95% CI 0.97–4.86, relative to ≤ 160 cm, *p* for trend = 0.032). Lastly, men were less likely to have the least positive experience (OR 0.56, 95% CI 0.33–0.96).

Age, BMI, general practice deprivation, smoking status, presence of comorbidities, and GORD Impact Scale were not found to have a significant impact in either the univariable or multivariable logistic regression analyses.

Since failed swallow at first attempt was such a strong predictor of the least positive patient experience, we additionally carried out univariable and multivariable analyses with it as the outcome. Results are shown in Table 2: in univariable analyses, only sex (male: OR 0.64, 95% CI 0.45–0.92, *p* = 0.02) was a significant predictor of a failed swallow at first attempt. However, there were no significant predictors of unsuccessful swallow when using backwards selection for the multivariable model.

**Table 2:** Logistic regression analyses examining predictors of a failed swallow at first attempt (n = 1445–1458 for unadjusted analyses)

Variables	Unadjusted odds ratio (95% CI)	*p*-value
*Sex*		
Female	1	
Male	0.64 (0.45–0.92)	0.02
*Age (years)*		
50–59	1	0.54
60–69	1.45 (0.85–2.47)	
70–79	1.38 (0.82–2.33)	
80 and over	1.17 (0.55–2.50)	
*Height*		
Up to 160 cm	1	0.06
> 160–170 cm	0.74 (0.49–1.13)	
> 170–180 cm	0.51 (0.31–0.84)	
> 180 cm	0.61 (0.31–1.21)	
*BMI*		
Underweight/normal weight (< 25)	1	0.55
Overweight (25.0–29.9)	1.14 (0.70–1.85)	
Obese (30 and over)	1.31 (0.80–2.15)	
*Deprivation level (IMD quintiles)*		
1—most deprived	1	0.19
2	0.73 (0.31–1.71)	
3	0.92 (0.46–1.84)	
4	1.38 (0.72–2.65)	
5—least deprived	1.39 (0.73–2.66)	
*Ever smoked*		
No	1	
Yes	0.75 (0.53–1.06)	0.11
*Alcohol intake*		
None	1	0.39
Occasional (once or twice per month)	0.75 (0.46–1.22)	
Weekends only	0.69 (0.42–1.12)	
Daily/most days	0.69 (0.42–1.13)	
*Education level*		
School up to 15–16 years of age	1.01 (0.65–1.58)	0.39
College or vocational school	1.30 (0.83–2.04)	
Professional training, university, or postgraduate degree	1	
*Comorbidities*		
No	1	
Yes	1.90 (0.98–3.68)	0.06
*State-trait anxiety inventory (STAI) score*		
Low/normal (20–36)	1	0.41
High (37–49)	1.24 (0.83–1.85)	
Very high (50–80)	1.36 (0.76–2.43)	
*GORD impact scale (week before appointment)*		
1 (low impact)	1	
> 1 (high impact)	1.25 (0.86–1.80)	0.24

*BMI* Body mass index; *IMD* Indices of deprivation; *GORD* Gastroesophageal reflux disease

*p-values: test for trend used for age, height, BMI, IMD quintiles, alcohol intake, education and STAI-6

## Discussion

The Cytosponge test is a safe and well tolerated method to screen for Barrett’s oesophagus that can be carried out in a primary care setting. The vast majority of BEST3 participants had a positive experience: the overall median IAPS score was 1.7 and only 4.7% had a score above 2.5. This study has given some insights into the factors that need attention to further improve the experience of patients undergoing the Cytosponge procedure. In adjusted analyses, anxiety and a failed swallow at first attempt were found to be strongly associated with the least positive experience, in addition to sex, height, alcohol intake and education level. A multivariable regression analysis did not identify any statistically significant predictors of unsuccessful swallow.

Around one third of the study cohort had high or very high anxiety scores just prior to the procedure and the adjusted odds of the least positive experience were 3–4 times higher in these groups than in those with low/normal anxiety levels. As the STAI-6 used to measure anxiety prior to the Cytosponge procedure did not specifically ask about the procedure, it is not clear whether these individuals felt anxious in general or about the procedure specifically. It may be that people who find swallowing tablets difficult, or the Cytosponge retrieval procedure particularly aversive, were more anxious about the procedure and also reported a less positive experience afterwards. It is also possible that people suffering from anxiety for reasons unrelated to the procedure were inclined to rate the experience more negatively. Identifying anxiety ahead of the procedure and discussing particular concerns with patients or giving them tips to help with swallowing the capsule might help improve their overall experience.

Secondly, having a failed swallow at first attempt was also a strong predictor of the least positive patient experience. As this is potentially modifiable, we attempted to identify predictors for it. However, when running a multivariable regression with backwards selection for unsuccessful swallow, none of the variables were significant predictors. Changes to the amount of water or way that water is offered (e.g. from a cup or with a straw) might reduce the risk of failed swallow attempts and could be tested in the future.

Drinking alcohol daily was associated with increased odds of having a less positive experience. According to some studies, alcohol consumption is directly related to increased symptoms of GORD [18–20]. Stomach acid could be very irritating to the lining of the oesophagus and prolonged exposure could lead to inflammation of the oesophagus, erosive oesophagitis, elevating the chances of discomfort and pain while pulling out the sponge contributing to least positive experience in our study. However, it is important to note that this study did not find any significant association between GORD Impact Scale and negative patient experience, so the pathway by which alcohol intake affected experience did not appear to be via GORD symptoms. It is therefore possible that alcohol consumption was a proxy for other predictors.

The participants with education from college or vocational school and school up to 15–16 years of age were less likely to have a negative experience as compared to those who were university educated; however, the test for a trend only reached borderline statistical significance. This finding is in line with past studies suggesting that a higher level of patient satisfaction is associated with lower educational level in outpatient care [21, 22]. Furthermore, one study in inpatient care found that patients with higher educational level seem to have a different view of the care given than patients with lower educational background [23], which could result in different expectations. A Danish study on breast cancer screening, on the other hand, found lower satisfaction amongst women with both low and high levels of education and highest satisfaction amongst women with medium education levels [24].

### Strengths and limitations

This study was part of a large pragmatic randomised controlled trial of the Cytosponge, in which nearly 1500 participants completed a 22-item patient experience survey (IAPS) covering all aspects of the Cytosponge appointment and procedure. Furthermore, detailed demographic and clinical information, in addition to anxiety data from the STAI-6 questionnaire, was available. This sample size and extensive data available allowed a more in-depth analysis looking specifically at predictors of the least positive experience. Moreover, the BEST3 trial took place in 109 general practice clinics in England, providing a socio-demographically diverse study population.

The main limitation of our study is that the IAPS, which had been adapted from flexible sigmoidoscopy, had only been validated by piloting with a small number of patients. However, the Cronbach’s alpha of 0.83 shows good internal reliability of this adaptation. A further limitation was that the follow-up questionnaire was completed 7–14 days after the procedure, which could have affected recall. This might have been the reason for not all participants completing all items in the questionnaire. Moreover, 30 individuals who had completed less than 15 items of the IAPS were excluded from the analysis, and individuals who did not successfully swallow a Cytosponge (N = 96), who may be expected to have had the least positive experience, were not asked to complete a questionnaire. The STAI-6 used to measure anxiety before the Cytosponge test and at follow-up did not specifically ask about the procedure, but measures state anxiety, which could be influenced by other factors. The STAI-6 was chosen as it is a short and reliable measure [18], which has been thoroughly validated. It was therefore very suitable for use in this set-up. Furthermore, comparing STAI-6 scores at baseline and follow-up found a significant difference in scores [15] indicating that the STAI-6 score at baseline was likely to have been affected by the upcoming Cytosponge test. As Index of Multiple Deprivation scores were not available for individual participants, the scores for their general practice clinic were assigned to each patient. The IMD is therefore only an approximation and not a representation of the true IMD of each individual. However, in the absence of individual IMD data, this is the best estimate available. Lastly, this analysis was not part of the primary or secondary outcomes of the BEST3 trial as defined in the trial protocol. However, a statistical analysis plan was developed prior to the analysis.

## Conclusion

This study, investigating factors contributing to a less positive experience of patients enrolled in the BEST3 trial, found that participants with high anxiety levels just before the appointment and those with failed swallows at first attempt were more likely to have a negative experience. Similarly, female sex, increased height, alcohol intake and obesity also predicted a less positive patient experience. Patient experience could therefore be improved by finding ways to reduce the risk of failed swallows and reassuring anxious patients.

## Supplementary Information

**Additional file 1.** Appendix.

## Data Availability

The trial protocol, statistical analysis plan, and statistical report are available via the University of Cambridge data repository (https://www.data.cam.ac.uk/repository). Datasets will be available from R Fitzgerald (rcf29@cam.ac.uk) on request.

## Abbreviations

GORD: Gastro-oesophageal reflux disease
IMD: Index of multiple deprivation
IAPS: Inventory to assess patient satisfaction
IQR: Interquartile range
OAC: Oesophageal adenocarcinoma(s)
OR: Odds ratio
STAI-6: State-trait anxiety inventory
TFF3: Trefoil factor 3
