# Supplementary material for: Predictors of the experience of a Cytosponge test: analysis of patient survey data from the BEST3 trial
Source: BMC Gastroenterol. 2023 Jan 10;23:7. doi: 10.1186/s12876-022-02630-1 (PMC9832657; doi:10.1186/s12876-022-02630-1)

Appendix

BEST3 Consortium

The Trial was managed by the Cancer Research UK & King's College London Cancer Prevention Trials Unit. The BEST3 consortium consists of the following:

Independent Data monitoring committee: Stephen Attwood (Chair), Max Parmar, Brendan Delaney.

Independent Trial steering committee: John de Caestecker (Chair), Wendy Atkin (in memoriam), Allan Hackshaw, Charles van Heyningen (patient representative), Tim Underwood

Cancer Prevention Trials Unit: Alberto Stella, Charlotte Saxby, Attila Lorincz, Naomi Turnbull, Jamie Doorbar, Georgia Mannion-Krase, Irene Kaimi (in memoriam)

Cambridge University Hospitals/Cambridge University: Staff at the Cambridge University Hospitals Human Research Tissue Bank, Mary Kasanicki, Stephen Kelleher, Louise Stockley, Tracy Assari, Sonakshi Kadyan, Victoria Hollamby, Katie Edwards CRN Eastern: Helen MacDonald, Viv Shaw, Heather Leishman, Holly Roper, Kate McCloskey, Helen Jung, Alex Phillips, Gosia Masjak-Newman, Kim Fell and the delivery team CRN Thames Valley and South Midlands: Helen Collins, Olga Zolle, and study delivery team CRN South West Peninsula: Pauline McGlone, Tania Crabb, Lauren Merrin and study delivery teams. CRN Wessex: Martine Cross, Alex Jones, Tom Simpson and study delivery team CRN North East and North Cumbria: Emma Murray and study delivery teams. CRN Yorkshire and Humber/Yorkshire practices: Study delivery teams. CRN North Thames: Andrew Perugia and study delivery team CRN East Midlands and University of Nottingham: Marie Thompson, Jen Dumbleton, Monique Morar and Nadia Frowd. Participating NHS trusts: Antonia Hardcastle, Debbie Carmichael, Fiona Maxton, Frances Farnworth, Elaine Baddeley

Supplementary Figure 1. Histogram showing the frequency distribution of the mean IAPS score for each participant.

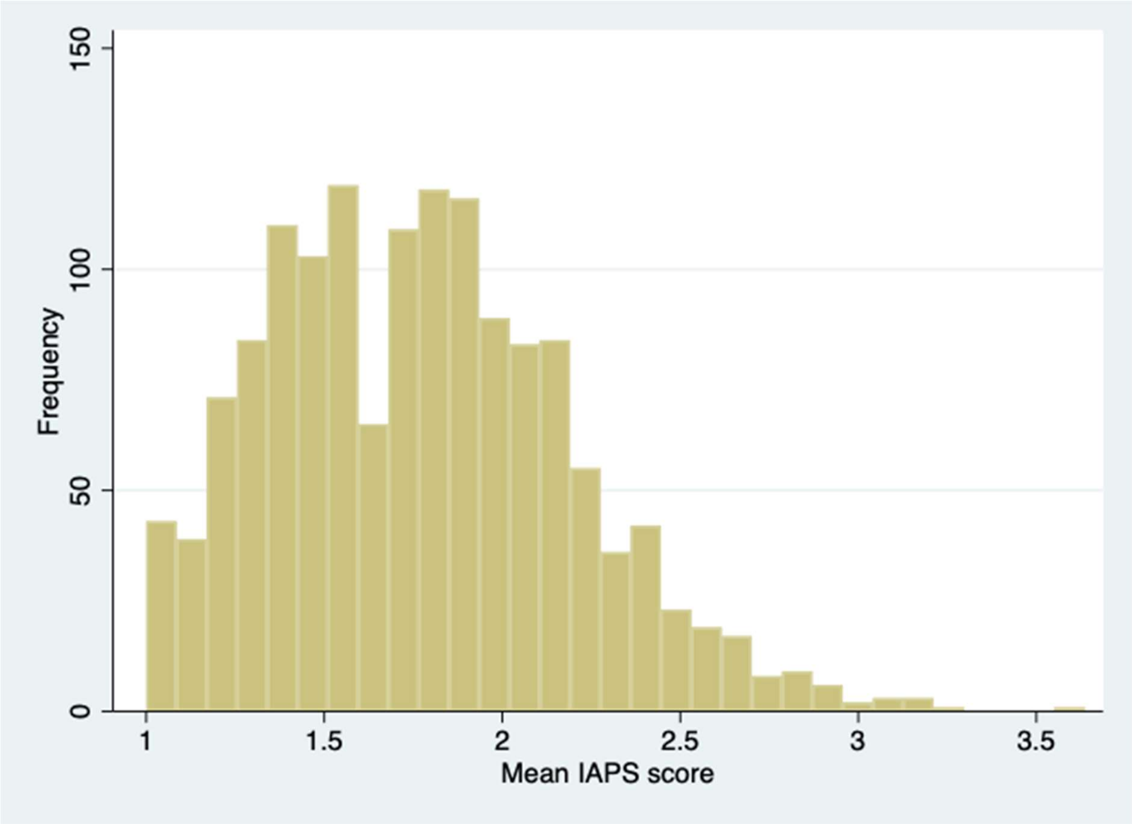

Supplement: Supplementary file 1 — Additional file 1. Appendix. [file 12876_2022_2630_MOESM1_ESM.pdf]
